# Book Reviews

**Published:** 1872-12-15

**Authors:** 


### Book Reviews.

Lessons in Physical Diagnosis. By Alfred L. Loomis, M.D., etc. Third edition. Revised and enlarged. New York: William Wood & Co. W. B. Keen & Cook, Chicago.

This is a work already well and favorably known to the profession. In the present edition the original text has been entirely revised and enlarged by the addition of five new lessons—three on the examination of the urine as applied to diagnosis, and two on the mechanical aids to diagnosis. These additions and revisions add greatly to the practical value of the work, and constitute it a much more complete treatise. The text is well illustrated by numerous wood cuts and engravings.

The Microscope and Microscopical Technology. A text book for Physicians and Students. By Heinrich Frey, M.D., Prof. of Medicine in Zurich, Switzerland. Translated and edited by George R. Cutter, M.D. From the fourth and last German edition. New York: Wm. Wood & Co. Chicago: For sale by W. B. Keen & Cook.

This is a volume of 650 pages, numerously and finely illustrated by some 340 engravings on wood. It is a work which, although it has been but a short time before the public, has already become an invaluable companion to those microscopists who are able to read it in the original. This English translation will, we feel confident, therefore meet with a most favorable reception from the large numbers of students, scientific and professional men to whom it has before been inaccessible. The table of contents embraces the following topics: "Theory of Microscopic; Apparatus for measuring and drawing; The Binocular Stereoscopic, and Polarizing Microscopes; Testing the Microscope; Use of the Microscope; The Preparation of Microscopic Objects; Fluid Media and Reagents; Methods of Stianing; Impregnation with Metals; The Drying and Freezing Processes; Method of Injecting; The Mounting and Arrangement of Objects; Blood, Lymph, Chyle, Mucus and Pus; Epithelium, Nails and Hair; Connective Tissue and Cartilage; Bones and Teeth; Muscles and Nerves; Vessels and Glands; Digestive Organs; Pancreas, Liver and Spleen; Respiratory Organs; Urinary Organs; Sexual Organs; Organs of Sense; Index; Price Lists of Microscope Makers."

